# Volumetric and End-Tidal Capnography for the Detection of Cardiac Output Changes in Mechanically Ventilated Patients Early after Open Heart Surgery

**DOI:** 10.1155/2019/6393649

**Published:** 2019-05-30

**Authors:** Ingrid Elise Hoff, Lars Øivind Høiseth, Knut Arvid Kirkebøen, Svein Aslak Landsverk

**Affiliations:** ^1^Norwegian Air Ambulance Foundation, P.O. Box 414 Sentrum, 0103 Oslo, Norway; ^2^Department of Anaesthesiology, Oslo University Hospital, P.O. Box 4956 Nydalen, 0424 Oslo, Norway; ^3^Section of Vascular Investigations, Oslo University Hospital, P.O. Box 4956 Nydalen, 0424 Oslo, Norway; ^4^Faculty of Medicine, University of Oslo, P.O. Box 1072 Blindern, 0316 Oslo, Norway

## Abstract

**Background:**

Exhaled carbon dioxide (CO_2_) reflects cardiac output (CO) provided stable ventilation and metabolism. Detecting CO changes may help distinguish hypovolemia or cardiac dysfunction from other causes of haemodynamic instability. We investigated whether CO_2_ measured as end-tidal concentration (EtCO_2_) and eliminated volume per breath (VtCO_2_) reflect sudden changes in cardiac output (CO).

**Methods:**

We measured changes in CO, VtCO_2_, and EtCO_2_ during right ventricular pacing and passive leg raise in 33 ventilated patients after open heart surgery. CO was measured with oesophageal Doppler.

**Results:**

During right ventricular pacing, CO was reduced by 21% (CI 18–24; *p* < 0.001), VtCO_2_ by 11% (CI 7.9–13; *p* < 0.001), and EtCO_2_ by 4.9% (CI 3.6–6.1; *p* < 0.001). During passive leg raise, CO increased by 21% (CI 17–24; *p* < 0.001), VtCO_2_ by 10% (CI 7.8–12; *p* < 0.001), and EtCO_2_ by 4.2% (CI 3.2–5.1; *p* < 0.001). Changes in VtCO_2_ were significantly larger than changes in EtCO_2_ (ventricular pacing: 11% vs. 4.9% (*p* < 0.001); passive leg raise: 10% vs. 4.2% (*p* < 0.001)). Relative changes in CO correlated with changes in VtCO_2_ (*ρ*=0.53; *p*=0.002) and EtCO_2_ (*ρ*=0.47; *p*=0.006) only during reductions in CO. When dichotomising CO changes at 15%, only EtCO_2_ detected a CO change as judged by area under the receiver operating characteristic curve.

**Conclusion:**

VtCO_2_ and EtCO_2_ reflected reductions in cardiac output, although correlations were modest. The changes in VtCO_2_ were larger than the changes in EtCO_2_, but only EtCO_2_ detected CO reduction as judged by receiver operating characteristic curves. The predictive ability of EtCO_2_ in this setting was fair. This trial is registered with NCT02070861.

## 1. Introduction

Haemodynamic deteriorations are frequent in many clinical situations but may initially be subtle and thus difficult to detect. Estimation of cardiac output (CO) or CO changes may help distinguish vasodilatation due to anaesthetic or sedative drugs from impairment of cardiac function or hypovolemia and help evaluate response to therapy. Thus, monitoring CO is recommended during major surgery [1] and circulatory failure [2]. Non- or minimally invasive CO monitoring methods are increasingly available but infrequently used. Factors that limit their use may be the need for extra equipment, operator dependency, or costs. Hence, simple, inexpensive, and preferably minimally invasive methods to monitor CO or changes in CO are needed.

Capnography is widely used in mechanically ventilated patients. During constant ventilation and metabolism and in the absence of lung disease, changes in exhaled carbon dioxide (CO_2_) reflect changes in pulmonary blood flow [3]. Exhaled CO_2_ can be expressed as end-expiratory partial pressure (EtCO_2_), or as volume eliminated CO_2_ per minute (VCO_2_) or per tidal volume (VtCO_2_). Volumetric capnography also provides information about pulmonary dead space and metabolism [3, 4]. Both volumetric and waveform capnography are recommended in the guidelines for mechanical ventilation [5] and several modern ventilators provide VtCO_2_ and VCO_2_ as well as EtCO_2_ [3, 6, 7].

Measurement of EtCO_2_ is included in the Advanced Cardiac Life Support guidelines [8], as EtCO_2_ reflects effective heart compressions and return of spontaneous circulation after cardiac arrest [9]. EtCO_2_ has also been shown to predict fluid responsiveness during passive leg raise (PLR) or after a fluid load [10–12] and is included in the 2014 guidelines on haemodynamic monitoring in circulatory shock [2]. However, the changes in EtCO_2_ following PLR or a fluid load are quite small (≈5%). This could limit their clinical use, as small changes are difficult to distinguish from random fluctuations. Some studies suggest that the changes in VCO_2_ following a preload challenge or increased positive end-expiratory pressure (PEEP) are larger [13, 14]. Good agreement has been shown between CO measurements by thermodilution and volumetric capnography in both animal [15, 16] and human studies [17, 18]. Recent clinical studies on the relationship between exhaled CO_2_ and CO have mainly focused on the prediction of fluid responsiveness, and EtCO_2_ has been investigated more often than VCO_2_ [11–14]. Few studies have investigated both VtCO_2_ and EtCO_2_ during moderate reductions in CO, although the detection and evaluation of decreases in CO is of major interest both perioperatively and during intensive care.

In the present study, we used right ventricular pacing (RVP) to induce moderate reductions in CO. RVP reduces CO by approximately 20% due to loss of atrial contribution [19] and dyssynchrony [20]. To the best of our knowledge, RVP has not previously been used as a model to investigate non- or minimally invasive CO monitoring methods.

The aim of this study was to investigate to what extent VtCO_2_ and EtCO_2_ reflect sudden moderate reductions in CO induced by RVP as well as sudden moderate increases in CO induced by PLR. We hypothesised that VtCO_2_ and EtCO_2_ would reflect changes in CO, that the changes in VtCO_2_ would be larger than the changes in EtCO_2_, and that the changes in CO, EtCO_2_, and VtCO_2_ would be correlated.

## 2. Methods

### 2.1. Patients

The study was approved by the regional ethics committee 02/07/2014 (REC South-East, 2013/1605) and registered in http://www.clinicaltrials.gov 02/23/2014 (NCT02070861), prior to patient enrolment. Forty adult patients scheduled for open coronary artery bypass surgery or aortic valve replacements were included from April 2014 to June 2015. Written informed consent was obtained prior to surgery. Patients with atrial fibrillation or ejection fraction <40% and patients in whom oesophageal Doppler placement was contraindicated were not included. The study was conducted in the cardiothoracic recovery unit of a university hospital 1-2 h after surgery. Patients were sedated with propofol 2-3 mg/kg/h according to departmental practice. They were haemodynamically stable prior to interventions, as evaluated by the attending cardiothoracic anaesthesiologist. Patients were ventilated in pressure-regulated volume control mode, tidal volumes 6–8 mL/kg predicted body weight, positive end-expiratory pressure 5–8 cmH_2_O, risetime 0.20 s, FiO_2_ as required for SpO_2_ >94%, and frequency 9–13 breaths/min, adjusted to obtain an EtCO_2_ between 32 and 38 mmHg before interventions (Evita Infinity C 500, Drägerwerk AG&Co, Lübeck, Germany). Ventilation and medication were kept constant during interventions (Table 1).

### 2.2. Data Acquisition and Analysis

EtCO_2_ was measured by using an analogue side-stream capnograph (Medlab CAP 10; Medlab GmbH, Stutensee, Germany) with infrared absorption technology and sampled at 400 Hz in SignalExpress 14.0.0 (National Instruments, Austin, Texas) after conversion in an analogue-to-digital converter (NIDAQPad-6015, National Instruments). Flow was measured continuously by Dräger Infinity ID with hot-wire anemometer technology. Haemodynamic data, including blood pressure obtained via a 20G catheter in the left radial artery, were downloaded from GE Solar 8000i (GE Healthcare, Chicago, Illinois, US) and analysed in a custom-made program (LabView 2010, National Instruments). CO was measured with oesophageal Doppler (DP-12 probe; Cardio Q; Deltex Medical, Chichester, UK), which continuously measures flow velocity in the descending aorta and thus rapid changes in stroke volume (SV) [21]. The Doppler probe was thoroughly fixed in the position that gave the best signal and maximum peak velocity of the aortic flow, and the signal was closely observed throughout experiments. SV measurements were downloaded beat-by-beat by the serial output.

### 2.3. Calculation of VtCO_2_

The volumetric capnograms were reconstructed from flow and EtCO_2_ curves for the calculation of VtCO_2_, as the VtCO_2_ and VCO_2_ values from the ventilator could not be extracted for offline analyses. Digital mainstream flow curves from the ventilator were continuously sampled on a laptop computer using Medibus software (Dräger, Drägerwerk AG&Co, Lübeck, Germany) and aligned with converted side-stream EtCO_2_ curves in a custom-made program in LabView, thereby accounting for the relative delay of 1–4 s of the side-stream capnogram [22]. The products of the flow and EtCO_2_ curves over time were integrated, giving VtCO_2_ for each respiratory cycle. Respiratory cycles containing nonpaced heartbeats during the RVP sequence were omitted.

### 2.4. Study Design

**Figure 1:**
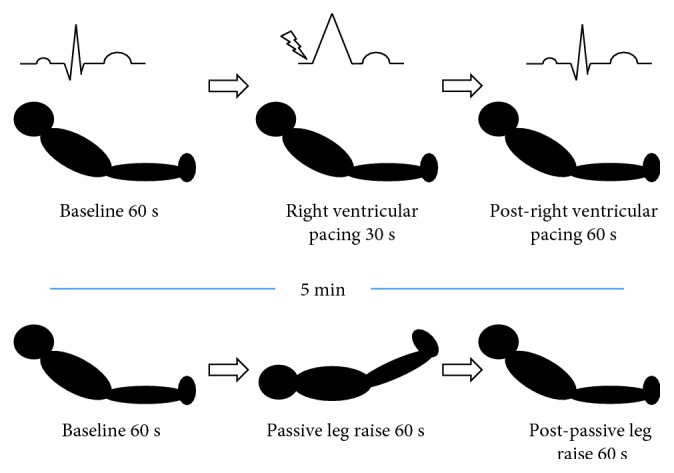
Study protocol. Sixty-second baseline measurements before 30 s of RVP and 60 s of PLR. The sequence of the interventions varied, minimum 5 min apart.

The experimental design is illustrated in Figure 1. Reduction in CO was obtained by right ventricular pacing. Epicardial pacemaker leads were established towards the end of surgery according to standard departmental practice. Pacing was induced by using an external pacemaker (Medtronic 5388 Dual Chamber Temporary Pacemaker, Medtronic, Minneapolis, USA). Pacing was performed by one of the department's cardiothoracic anaesthesiologists similarly to the pacemaker test routinely performed in patients who require postoperative pacing. Pace rate was set marginally higher than the patient's own heart rate in order to prevent spontaneous beats, but as low as possible to prevent increased heart rate from offsetting the intended reduction in SV. Calculations were made from measurements obtained during 30 s of uninterrupted RVP, approximately 6 breaths. Increases in CO were induced by PLR, where the patient's position was altered from semirecumbent to horizontal with legs elevated 45°. This manoeuvre represents an endogenous and reversible fluid challenge of approximately 300 ml, with maximal volume effect during the first minute [23]. Thus, calculations were based on measurements from the initial 60 s after leg raise, approximately 12 breaths. Interventions were minimum 5 min apart to ensure return to baseline (BL) before new measurements. Sixty seconds of BL were recorded before and after each intervention with calculations based on BL measurements before interventions.

### 2.5. Data Analysis

CO, VtCO_2_, and EtCO_2_ were normally distributed assessed by the Shapiro–Wilk test. The effect of RVP and PLR on each variable and the difference between changes in VtCO_2_ and EtCO_2_ were tested using paired *t*-tests. The correlations between the relative changes from baseline to interventions in CO, VtCO_2_, and EtCO_2_ were analysed using the Spearman test of correlation, as these changes were mainly not normally distributed. Precision was calculated from the baseline sequence as 1.96 × √(within-subject mean square) in a one-way ANOVA with subjects as factors [24] and presented relative to the grand mean value. We considered the average of 30 s a clinically reasonable measurement unit and divided the breath-to-breath precision by √6 (corresponding to 12 breaths/min). Least significant change (LSC) was calculated as √2 × precision [25]. Analyses were performed in SPSS Statistics 24 (IBM, Armonk, New York, USA). We originally planned the presented analyses as part of a study comparing two different CO measurement devices, and sample size was calculated for the intended comparison. However, due to technical difficulties, that part of the study had to be aborted as we could not guarantee the validity of the data. No post hoc power analysis was undertaken for the present analyses, but confidence intervals are presented, according to the recommendations in the CONSORT guidelines [26]. A change in CO of 15% was considered clinically significant. Based on the results of a previous study, this corresponds to changes of approximately 7.5% in VtCO_2_ and 3.8% in EtCO_2_ [13]. Areas under the receiver operating characteristic (ROC) curves for EtCO_2_ and VtCO_2_ were calculated and compared in MedCalc Software 18.11 (MedCalc Software bvba, Ostend, Belgium). Their discriminative value was evaluated by their ability to detect a change in CO of 15%. *p* values <0.05 were considered statistically significant and all tests were two-tailed. Calculations and analyses were performed without blinding.

## 3. Results

**Figure 2:**
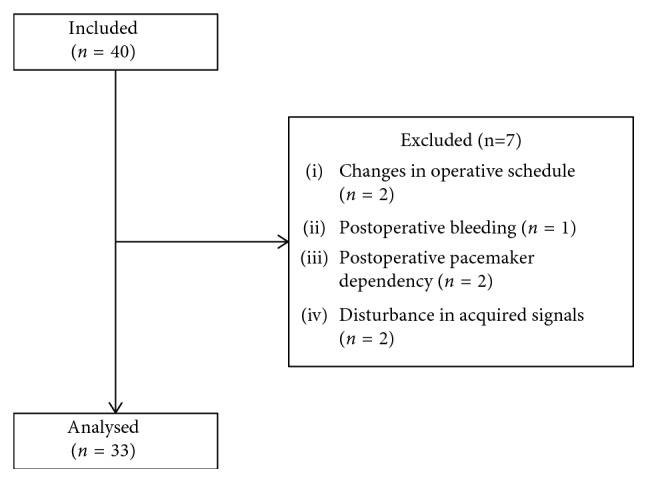
Flow chart inclusion.

Two patients were included, but not studied, due to changes in the operative schedule. One patient was excluded due to postoperative bleeding and two because they were pacemaker-dependent after surgery. Two patients were excluded because of disturbances in the acquired data signals. Thus, 33 patients (29 men, 4 women) completed the study (Figure 2).

**Figure 3:**
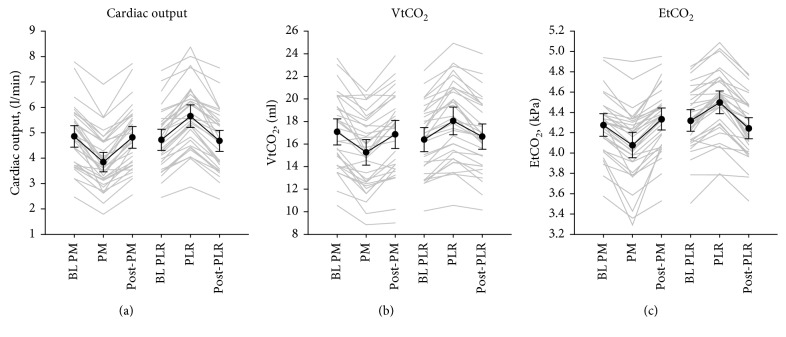
Lineplot. Individual (grey) and mean (black) values with 95% confidence intervals for CO, VtCO_2_, and EtCO_2_ before, during, and after interventions. CO = cardiac output; EtCO_2_ = end-tidal carbon dioxide; VtCO_2_ = exhaled carbon dioxide per tidal volume; BL = baseline; RVP = right ventricular pacing; PLR = passive leg raise.

**Figure 4:**
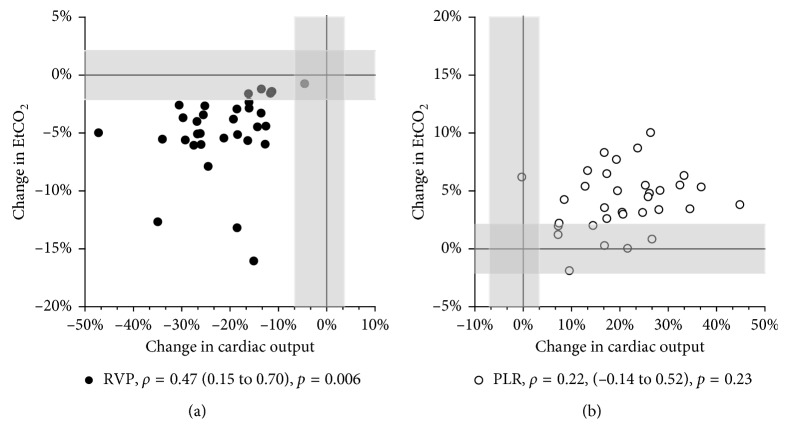
Scatterplot EtCO_2_. Correlation between mean relative changes in CO and EtCO_2_ from BL to RVP and PLR, respectively. Least significant changes for CO and EtCO_2_ are indicated with shadows. CO = cardiac output; EtCO_2_ = end-tidal carbon dioxide; VtCO_2_ = exhaled carbon dioxide per tidal volume; BL = baseline; RVP = right ventricular pacing; PLR = passive leg raise; *ρ* = Spearman's rho with confidence intervals.

Figure 3 shows individual and mean values at all 6 measurement points. For all variables, there were statistically significant reductions in mean scores from BL to RVP and statistically significant increases from RVP to BL and from BL to PLR (Table 2, Figure 3). The confidence intervals of the line plots in Figure 3 indicate that the study was not underpowered for the presented analyses. From BL to RVP, CO was reduced by 21.0% (CI 18–24; *p* < 0.001), VtCO_2_ by 11% (CI 7.9–13; *p* < 0.001), and EtCO_2_ by 4.9% (CI 3.6–6.1; *p* < 0.001). Relative changes in CO correlated significantly with changes in both VtCO_2_ (*ρ*=0.53; *p*=0.002) and EtCO_2_ (*ρ*=0.47; *p*=0.006) (Figure 4). From BL to PLR, CO increased by 21% (CI 17–24; *p* < 0.001), VtCO_2_ by 10% (CI 7.8–12; *p* < 0.001), and EtCO_2_ by 4.2% (CI 3.2–5.1; *p* < 0.001). None of these changes were significantly correlated (Figure 4). Overall, the changes in VtCO_2_ were significantly larger than the changes in EtCO_2_ (from BL to RVP, 11% vs. 4.9% (*p* < 0.001); from BL to PLR, 10% vs. 4.2% (*p* < 0.001)).

**Figure 5:**
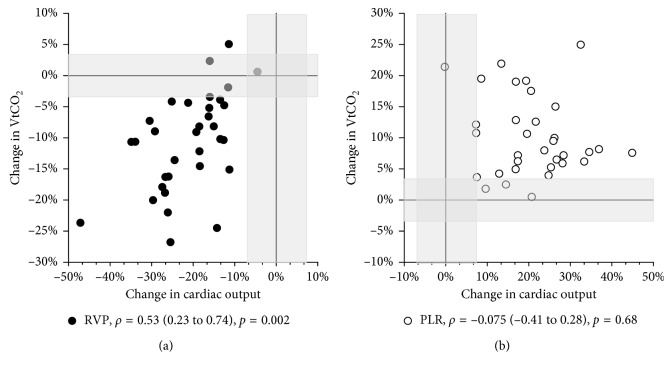
Correlation between mean relative changes in CO and VtCO_2_ from BL to RVP and PLR, respectively. Least significant changes for CO and VtCO_2_ are indicated with shadows. Dots are for RVP; circles are for PLR. CO = cardiac output; EtCO_2_ = end-tidal carbon dioxide; VtCO_2_ = exhaled carbon dioxide per tidal volume; BL = baseline; RVP = right ventricular pacing; PLR = passive leg raise; *ρ* = Spearman's rho with confidence intervals.

Precision and LSC for 30 s baseline measurements were 4.8% and 6.9%, respectively, for CO, 2.4% and 3.4% for VtCO_2_, and 1.5% and 2.1% for EtCO_2_. Thus, all mean changes seen after the interventions were larger than the LSC. The LSC for CO, VtCO_2_, and EtCO_2_ are indicated in Figures 4 and 5, respectively. According to the scatterplots during RVP, a reduction in VtCO_2_ and EtCO_2_ larger than the LSC implicated a reduction in CO of more than 11% for all subjects.

**Figure 6:**
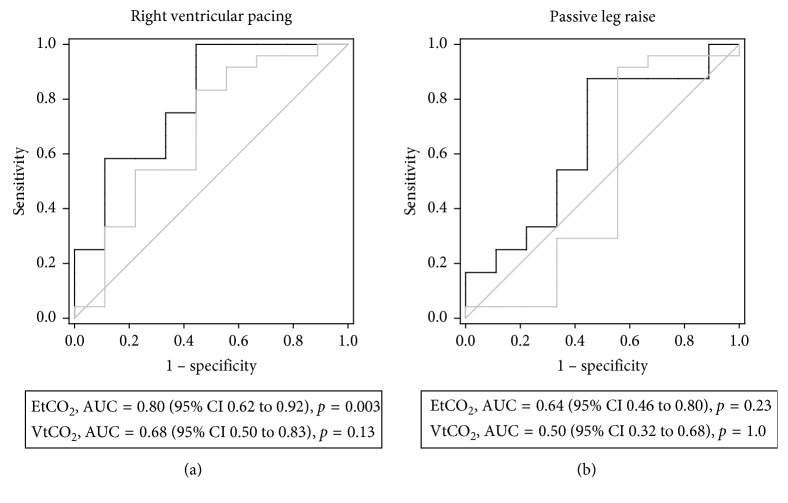
ROC-plot. Receiver operating characteristic plots of VtCO_2_ and EtCO_2_ during right ventricular pacing and passive leg raise, respectively. EtCO_2_ = end-tidal carbon dioxide; VtCO_2_ = exhaled carbon dioxide per tidal volume; AUC = area under the curve.

ROC-plot analyses are shown in Figure 6. The best discriminative ability was found for EtCO_2_ (AUC 0.80; 95% CI 0.62–0.92, *p*=0.003) during RVP, whereas the ROC curve for VtCO_2_ was not significantly different from 0.5. Neither EtCO_2_ nor VtCO_2_ was able to discriminate changes in CO during PLR.

## 4. Discussion

The main findings of this study were that VtCO_2_ and EtCO_2_ tracked sudden moderate reductions in CO. Both reductions and increases in CO with RVP and PLR coincided with reductions and increases, respectively, in EtCO_2_ and VtCO_2_ (Figure 3). The magnitudes of the changes, however, were only correlated when CO was reduced, and correlations were modest (Figures 4 and 5). According to the ROC analyses, only EtCO_2_ was able to discriminate changes in CO using a threshold of 15% change and only the reduction during RVP (Figure 6).

Young et al. [13] found VCO_2_ superior to EtCO_2_ for predicting fluid responsiveness in the PLR model, and the changes in VCO_2_ were substantially larger than the changes in EtCO_2_. Tusman et al. [14] showed that a reduction in VCO_2_ following an increase in PEEP predicted fluid responsiveness with better sensitivity and specificity than EtCO_2_. In our study, the changes in CO during RVP appear to be slightly stronger correlated with the changes in VtCO_2_ than with the changes in EtCO_2_. Precision was better for EtCO_2_ than for VtCO_2_, but this did not outweigh the larger effect of changes in CO on VtCO_2_. The ROC analyses, using a threshold of 15%, indicate a stronger discriminative ability for EtCO_2_ than VtCO_2_, which appears contradictory to the previously mentioned findings. However, the criterion value giving the maximal Youden index for EtCO_2_ was low (Table 3), limiting its use as a clinical cutoff value. There are also some limitations to the ROC analysis associated with the dispersion of predictor values in the population which is investigated. These limitations are previously described [27] and highlighted in a recent review [28] and should be considered when comparing AUC values from different studies.

In the studies by Monge García et al. [10] and Monnet et al. [11], EtCO_2_ predicted fluid responsiveness with higher sensitivity and specificity than arterial pulse pressure, and Jacquet-Lagreze et al. [12] found the same when comparing EtCO_2_ to MAP. These findings were confirmed in a recent study by Lakhal et al. [29], who in addition found that EtCO_2_ assessed fluid responsiveness better than changes in systolic blood pressure and femoral blood flow did. In summary, while EtCO_2_ has been found superior to other widely used noninvasive indices, newer studies suggest that VCO_2_ and VtCO_2_ could be superior to EtCO_2_. In the present study, the changes in VtCO_2_ were substantially larger than the changes in EtCO_2_ following a given change in CO, and correlations were similar. However, given that a diagnostic ability was demonstrated only for EtCO_2_, the results do not support the superiority of VtCO_2_ over EtCO_2_. In some of the studies, VCO_2_ and EtCO_2_ were also found superior to pulse pressure variations (PPVs) or stroke volume variations (SVVs) in the presence of arrhythmia [29] or tidal volumes <8 mL/kg [14, 29]. This is explained by the fact that PPV and SVV are validated for the prediction of fluid responsiveness mainly in patients with tidal volumes ≥8 mL/kg and without arrhythmia [30, 31]. However, as protective ventilation becomes the norm, it is noteworthy that the same restrictions do not seem to apply for EtCO_2_ or VtCO_2_.

The physiologic relationship between exhaled CO_2_ and CO in dynamic states is previously described [15, 32]. Reduced pulmonary perfusion leads to reduced CO_2_ transport to the lungs and increased alveolar dead space; both resulting in reduced CO_2_ elimination. With increased pulmonary perfusion, more CO_2_ is brought to the lungs, underperfused lung tissue is recruited, and CO_2_ elimination is increased. Although reports of the nature of the relationship between exhaled CO_2_ and CO differ [18, 32, 33], several studies have found significant correlations between changes in CO and changes in EtCO_2_ after PLR [10, 11]. We believe there are mainly two reasons why there were no correlations between EtCO_2_, VtCO_2_, and CO during PLR in our study. Firstly, previous studies investigated patients with circulatory failure, whereas our cohort was haemodynamically and metabolically stable. The relationship between changes in CO and exhaled CO_2_ is stronger during unstable circulatory states, e.g., in patients with reduced CO [6]. In steady states, exhaled CO_2_ mainly depends on CO_2_ production. Lung perfusion and ventilation/perfusion ratio will be affected only marginally, if at all, by an increase in CO of 20% in euvolemic patients who are adequately ventilated. This is in line with the findings of Ornato et al [32], who in an animal study demonstrated that the correlation between changes in CO and changes in EtCO_2_ decreased as CO reached normal or supranormal values, when pulmonary flow no longer represents a limitation to the CO_2_ elimination via the lungs. By contrast, we observed significant correlations between the relative reductions in CO, VtCO_2_, and EtCO_2_ when CO was decreased during the RVP sequence, even though the change in CO was of similar magnitude. Secondly, the mean relative increase in EtCO_2_ during PLR in our study was 4.2%, which is smaller than in previous studies which have reported an increase of >5%. As these studies were designed to study fluid responsiveness, EtCO_2_ was recorded during the maximal haemodynamic changes following PLR. We sampled CO, EtCO_2_, and VtCO_2_ over 1 min of PLR, and although the main preload increase is likely to take place within that minute, the time span includes lower values that dilute this effect. Also, it is possible that the position change during the PLR manoeuvre could affect CO_2_ elimination by other mechanisms than the preload increase. This could have influenced the results. In a postoperative setting with haemodynamically stable patients, the detection of a sudden decrease in CO, e.g., due to bleeding, is arguably more relevant than the prediction of preload responsiveness.

In the absence of CO monitoring, MAP is often used for haemodynamic assessment. As MAP is highly influenced by vascular resistance [34], it may be affected by anaesthetics, pain, hypovolemia, and hypothermia. Hypotension occurs frequently in the operating room or intensive care unit and can be due to a number of causes. By also considering changes in EtCO_2_ or VtCO_2_ in cases of decreasing blood pressure, the clinician may be aided in their therapeutic decisions.

### 4.1. Methodological Considerations

As departmental logistics had to be considered during data acquisition, the order of interventions varied in a nonrandomised fashion. The possibility of carryover effects was minimised by ensuring sufficient time between all interventions but cannot be excluded.

There was a departmental change in monitoring equipment during the study, and the available software did not allow export of invasive blood pressure data from the new monitors to the computer. Thus, MAP measurements were retrospectively obtainable from 21 patients only. This represents a limitation to the study.

CO had to be monitored continuously as changes in CO induced by RVP and PLR are rapid and transient. However, CO measurement with oesophageal Doppler has some limitations. Measurements are based on assumptions regarding the diameter of the aorta, angle of insonation, and fraction of CO that enters the descending aorta [35]. As we measured relative changes, the results would only have been affected if the assumed variables changed during experiments. Aortic diameter has been shown to change after a fluid load [36], and we cannot exclude a similar effect after PLR. These limitations suggest that oesophageal Doppler may perform better as a monitor of CO trends than of absolute values. This may also explain why some patients in the present study demonstrated rather low CO values despite being assessed as haemodynamically stable at baseline.

For the description of metabolism, exhaled CO_2_ is mostly expressed as VCO_2_, whereas both VCO_2_ and VtCO_2_ have been used to describe the relationship between exhaled CO_2_ and circulation [13, 15, 37]. We measured VtCO_2_ to enable a direct comparison with EtCO_2_, which is also measured breath-to-breath. As ventilation was kept constant throughout experiments, the choice of VtCO_2_ over VCO_2_ should not affect the results, which may therefore be seen in relation to previous studies investigating VCO_2_. The absolute changes in VtCO_2_ are small. However, they are significantly larger than the corresponding changes in EtCO_2,_ which use is already implemented in guidelines for haemodynamic evaluation. Modern ventilators display updated VCO_2_ values after each breath. For clinical use, changes in VCO_2_ may be easier to detect than changes in VtCO_2_, as they appear larger.

Any form of ventilation/perfusion mismatch may affect the relationship between CO and exhaled CO_2_ [38]. Other investigators have therefore excluded patients with pulmonary dysfunction [14, 18]. Only three of our patients (9.1%) had been diagnosed with chronic obstructive pulmonary disease. However, it is possible that some had undiagnosed lung disease or postoperative pulmonary dysfunction which may have affected our results.

As mechanical ventilation alters pulmonary physiology and haemodynamics [39], further studies are necessary to elucidate the performance of VtCO_2_ and EtCO_2_ in spontaneously breathing patients.

## 5. Conclusion

VtCO_2_ and EtCO_2_ tracked reductions in cardiac output, but correlations between the changes were modest. Judged by receiver operating characteristic curves, a CO reduction was only detected by EtCO_2_. Further studies are warranted to establish the role of exhaled CO_2_ as a clinical tool for detecting cardiac output changes in this setting.

## Figures and Tables

**Table 1:** Patient characteristics.

Variable	Mean (SD)
Age (years)	65 ± 9
Gender, male/female, *n* (%)	29 (88)/4 (12)
Height (cm)	177 ± 8
Weight (kg)	87 ± 12
Tidal volume (mL·kg^−1^ predicted body weight)	6.8 ± 1.1
PIP (cmH_2_O)	23 ± 2
Respiratory rate (min^−1^)	12 ± 1
Procedure CABG/AVR, *n* (%)	26 (79)/7 (21)
COPD, *n* (%)	3 (9)
Patients receiving nitroglycerin 0–2.5 *μ*g·kg^−1^·min^−1^, *n* (%)	12 (36)
Patients receiving nitroprusside 0.8–1.8 *μ*g·kg^−1^·min^−1^, *n* (%)	2 (6)
Patients receiving norepinephrine 0.02 *μ*g·kg^−1^·min^−1^, *n* (%)	1 (3)
Patients receiving amiodarone 900 mg·24·h^−1^, *n* (%)	1 (3)

Data are mean ± SD unless otherwise stated. PIP = peak inspiratory pressure; PEEP = positive end-expiratory pressure; CABG = coronary artery bypass grafting; AVR = aortic valve replacement; COPD = chronic obstructive pulmonary disease.

**Table 2:** Haemodynamic data at baseline and during right ventricular pacing and passive leg raise.

	BL	VP	*p* value	BL	PLR	*p* value
CO (l/min)	4.86 ± 1.20	3.84 ± 1.08	<0.001	4.72 ± 1.17	5.65 ± 1.26	<0.001
SV (mL/min)	71 ± 20	54 ± 17	<0.001	69 ± 17	81 ± 19	<0.001
VtCO_2_ (ml)	17 ± 3	15 ± 3	<0.001	16 ± 3	18 ± 4	<0.001
EtCO_2_ (kPa)	4.3 ± 0.3	4.1 ± 0.4	<0.001	4.3 ± 0.3	4.5 ± 0.3	<0.001
MAP (mmHg)^*∗*^	72 ± 6	62 ± 9	<0.001	72 ± 9	78 ± 8	<0.001
HR (beats/min)	69 ± 10	73 ± 10	<0.001	70 ± 10	71 ± 10	0.015

Data are presented in mean ± SD. BL = baseline; RVP = ventricular pacing; PLR = passive leg raise; CO = cardiac output; SV = stroke volume; VtCO_2_ = exhaled carbon dioxide per tidal volume; EtCO_2_ = end-tidal carbon dioxide; MAP = mean arterial pressure (^*∗*^measurements from 21 patients); HR = heart rate.

**Table 3:** Sensitivity, specificity, likelihood ratios, and predictive values set at criterion value giving maximal Youden index. The prevalence of responders and nonresponders was set to the ratio in the sample when calculating predictive values.

	AUC (95% CI)	Criterion (%)	Specificity (%)	Sensitivity (%)	+LR	−LR	PPV (%)	NPV (%)
VtCO_2_ at RVP	0.68 (0.50 to 0.83)	5.2	83	56	1.9	0.3	83	56
EtCO_2_ at RVP	0.80 (0.62 to 0.92)	1.7	100	56	2.3	0.0	86	100
VtCO_2_ at PLR	0.50 (0.32 to 0.68)	4.2	92	44	1.7	0.19	82	67
EtCO_2_ at PLR	0.64 (0.46 to 0.80)	2.2	88	56	2.0	0.23	84	63

AUC = area under the curve; +LR = positive likelihood ratio; −LR = negative likelihood ratio; PPV = positive predictive value; NPV = negative predictive value; VtCO_2_ = exhaled carbon dioxide per tidal volume; EtCO_2_ = end-tidal carbon dioxide; RVP = ventricular pacing; PLR = passive leg raise.

## Data Availability

The data used to support the findings of this study are restricted by Oslo University Hospital in order to protect patient privacy. Pseudonymised data are available from the corresponding author for researchers who meet the criteria for access to confidential data.
